# Exploring the mechanism of Buyang Huanwu decoction in the treatment of lumbar disc herniation based on network pharmacology and molecular docking

**DOI:** 10.1097/MD.0000000000029534

**Published:** 2022-08-12

**Authors:** Yong Gu, Haijia Zhu, Xiaojian Wang, Shanxing Zhang, Peijian Tong, Shuaijie Lv

**Affiliations:** a Zhangjiagang TCM Hospital Affiliated to Nanjing University of Chinese Medicine, Suzhou, China; b Zhejiang Chinese Medical University, Hangzhou, China; c The First Affiliated Hospital of Zhejiang Chinese Medical University, Hangzhou, China.

**Keywords:** active compounds, Buyang Huanwu decoction, lumbar disc herniation, molecular docking, molecular mechanism

## Abstract

Buyang Huanwu decoction (BYHWD), as one of the traditional Chinese medicine formulas, is widely used in the clinical treatment of lumbar disc herniation (LDH) with curative effect. It has the characteristics of multi-component, multi-target, and mutual synergy, but the mechanism of action is often unclear. It needs some research to explore the molecular mechanism of BYHWD in the treatment of LDH based on network pharmacology and molecular docking. Screen the active compounds of BYHWD and predict drug-related gene/protein targets, which could determine the specific target of BYHWD in the treatment of LDH. Construct the “Drugs-Compounds-Targets” network and search for the core targets. Use Gene Ontology functional enrichment analysis, Kyoto Encyclopedia of Genes and Genomes pathway enrichment analysis, and molecular docking verification to explore the possible molecular mechanism. Eighty-two effective compounds and 666 targets of BYHWD, 187 targets for LDH treatment, and 20 core candidate targets were excavated. A total of 3414 entries were identified by Gene Ontology enrichment analysis, 173 related signal pathways were identified by Kyoto Encyclopedia of Genes and Genomes enrichment analysis, and 5 core compounds were identified by molecular docking, which had a good affinity with core genes STAT3, JUN, AKT1, MAPK1, RELA, and PIK3CA. BYHWD may play the role of analgesic and improving function by synergistic anti-inflammatory and analgesic compounds, regulating cell metabolic differentiation, regulating immunity, and anticoagulation. BYHWD in the treatment of LDH may play a role in analgesia and improve function through multiple signaling pathways, including PI3K-Akt, mitogen-activated protein kinase, tumor necrosis factor, and interleukin-17. The PI3K-Akt signaling may be one of the key mechanisms.

## 1. Introduction

Lumbar disc herniation (LDH) is a clinical syndrome in which the annulus fibrosus of the lumbar disc is partially or completely ruptured due to various reasons, and nucleus pulposus tissue protrudes backward from the rupture opening, stimulating or compressing nerve root and cauda equina nerve. With the aging of population and the change of lifestyle, the incidence of LDH has increased significantly and tends to be younger.^[1]^ LDH patients showed a variety of clinical manifestations such as low back pain, numbness, fatigue of lower limbs, etc. In severe cases, cauda equina nerve injury occurs, resulting in defecation disorder and even paralysis. The occurrence and progress of LDH have a serious impact on the quality of life, and it also brings serious economic pressure to patients’ families due to the high disability rate. The treatment scheme of LDH includes conservative treatment and surgical treatment. Studies have shown that surgical treatment is more effective for patients with severe nerve root compression and inflammation symptoms, or without improvement after 3 to 6 months of non-surgical treatment.^[2]^ However, conservative treatment is still the first choice for LDH.^[3]^ Therefore, the development of complementary and alternative therapies for LDH is crucial.

**Figure 1.**
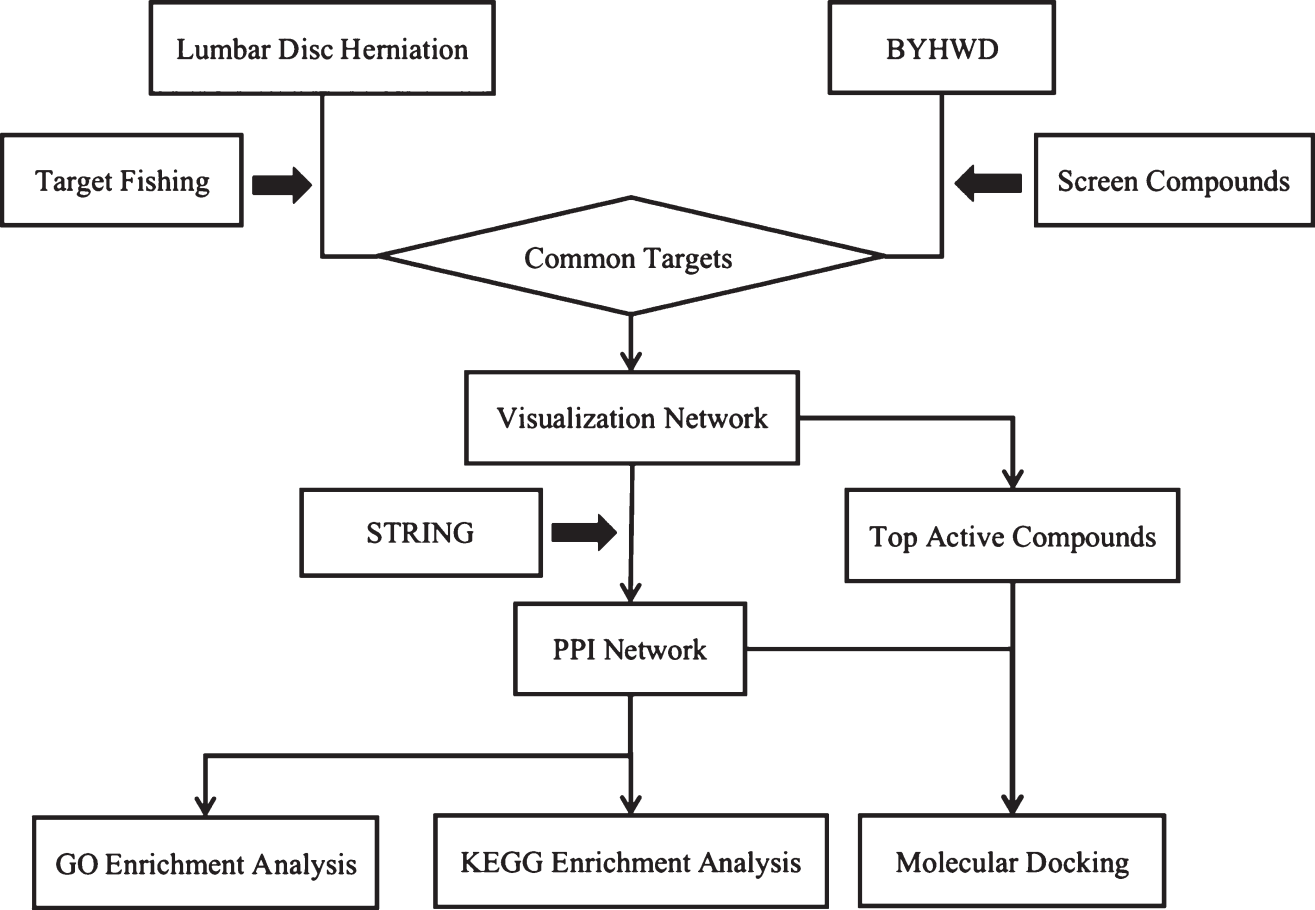
The whole framework based on an integration strategy of network pharmacology.

Traditional Chinese medicine (TCM), one of the complementary therapies, has achieved significant effects in treating LDH and helped broaden the ideas of therapeutic approaches for LDH. Buyang Huanwu decoction (BYHWD), as one of the TCM formulas, including Huang-Qi, Dang-Gui, Tao-Ren, Hong-Hua, Chi-Shao, Chuan-Xiong, and Di-Long, is widely used in the clinical treatment of LDH with curative effect.^[4,5]^ The TCM formula such as BYHWD has the characteristics of multi-component, multi-target, and mutual synergy, but the mechanism of action is often unclear. Network pharmacology screens synergistic multiple compounds from TCM formula in a high-throughput manner and explains the combination rules and network regulatory effects of TCM formulas.^[6,7]^ We analyzed key targets and signal pathways of BYHWD in the treatment of LDH and explored its possible molecular mechanism based on network pharmacology, bioinformatics, and molecular docking (Fig. 1).

## 2. Materials and Methods

### 2.1. Database and software

#### 2.1.1. Database.

Database and Analysis Platform of Systematic Pharmacology of Traditional Chinese Medicine (TCMSP Database, https://tcmspw.com/tcmsp.php/); PubChem Database (https://pubchem.ncbi.nlm.nih.gov/); Swiss ADME Database (http://www.swissadme.ch/); Swiss Target Prediction Database (http://swisstargetprediction.ch/); Gene Cards Database (https://www.genecards.org/); Database of Online Mendelian Inheritance in Man (OMIM Database, https://www.genecards.org/); Pharm GKB Database (https://www.pharmgkb.org/); DisGeNET (https://www.disgenet.org/); The Genetic Association Database (GAD Database, https://geneticassociationdb.nih.gov/); Uniprot Database (https://www.uniprot.org/); STRING 11.0 (https://string-db.org/); and Protein Date Bank (PDB) (https://www.rcsb.org/).

#### 2.1.2. Software.

Microsoft Office 365 Excel (Microsoft) was used for text, document collection, and data collation; R 4.0.3 software (R Development Core Team) and associated toolkits were used to analyze and complete visualization; Cytoscape 3.8.2 (National Institute of General Medical Sciences (NIGMS)) and related plug-ins are used to build visual networks; ChemBio 3D Ultra 14.0.0.117 (PerkinElmer) was used to convert the 2D structure to 3D form; PyMOL 2.4.0 (Schrodinger) was used to remove small-molecule ligands and water molecules and visualize them; Auto Dock Tools 1.5.6 (The Scripps Research Institute) software was used for hydrogenation and other processes; Vina 1.1.2 (Dr. Oleg Trott from The Scripps Research Institute) was used to process molecular docking and analyze binding activity; and GraphPad Prism 8.3.0 (GraphPad Software) was used to build heatmaps. This study was approved by the Ethics Committee of the First Affiliated Hospital of Zhejiang Chinese Medical University.

### 2.2. Main active ingredients and targets of BYHWD

Each Chinese herb included in BYHWD (Huangqi, Danggui, Taoren, Honghua, Chishao, Chuanxiong, and Dilong) was used as keywords for retrieval in the TCMSP database. The effective compounds of Chinese herbs were obtained from “Ingredients” and screened according to 2 criteria, “oral bioavailability ≥30%” and “drug-like index ≥0.18”.^[8,9]^ We collected potential protein targets for each drug in “Related Targets”. For drugs that could not be retrieved in the database, their ingredients were obtained by searching the literature. We downloaded their structures from the PubChem database and entered them into the Swiss ADME platform. In the results of “pharmacokinetics”, “GI absorption: high” was set as the precondition, and the standard that 2 or more other indicators display “yes” was used to screen compounds.^[10,11]^ We subsequently obtained the compound corresponding acting target proteins by inputting the compounds in the Swiss Target Prediction platform and eliminated items with “probability” as “0”. Finally, we filtered the items by “reviewed” in the UniProt database with the species selection “Homo sapiens (human)” to normalize the resulting protein targets.

### 2.3. Acquisition of disease targets for LDH

We selected “lumbar disc herniation” as the keyword and screened disease-related targets in GeneCards, OMIM, PharmGKB, DisGENET, and GAD databases. The Venn diagram was drawn by R 4.0.3 software and packages. We eliminated the duplicate items in Excel and obtained unique LDH-related targets.

### 2.4. Construction and analysis of visualized network

We used R software to match and map drug prediction targets to disease targets. We drew the Venn diagram of the “drugs-disease targets” intersection and extracted the common targets—the key targets of BYHWD in treating LDH. Then we used Cytoscape to form a “Drugs-Compounds-Targets” visualization network.

### 2.5. Construction of protein-protein interaction network

We uploaded the key proteins obtained from network analysis to the online STRING 11.0 (ELIXIR infrastructure) database, setting the type as “homo sapiens”, setting the confidence threshold as “highest confidence (0.900)”, hiding the disconnected nodes, and kept other setting parameters unchanged to obtain protein-protein interaction (PPI) network.

### 2.6. Obtain key genes from PPI network

We imported the PPI network into Cytoscape and CytoNCA network topology analysis plug-in was used to calculate the betweenness centrality, closeness centrality, degree centrality, eigenvector centrality, local average connectivity, and network centrality. We screened out the nodes whose above indexes were greater than the median of all gene nodes of each index by R software. Then we created the sub-network, and key genes were calculated and screened again.

### 2.7. Enrichment analysis

We used R software “ClusterProfiler” package to perform Gene Ontology (GO) function enrichment analysis at the different biological process, cellular component, and molecular function levels of key targets. Then Kyoto Encyclopedia of Genes and Genomes (KEGG) pathway enrichment analysis was carried out at key targets.

### 2.8. Verification of molecular docking

We downloaded the active ingredients’ 2D structure of small molecule ligands from the PubChem database, and imported them into ChemBio3D Ultra, then calculated and set them as the 3D structure with minimum free energy. We downloaded 3D structure files of core target proteins from the PDB database and imported them into PyMOL software to remove small-molecule ligands and water molecules, then we used Auto Dock Tools software for hydrogenation. Finally, we performed molecular docking of ligand and receptor via Vina, analyzed binding activity, and constructed the heatmap with Prism.

## 3. Results

### 3.1. Active ingredients and targets of BYHWD

According to the TCMSP database, the effective compounds of Astragalus Mongholicus, Angelica Sinensis, Radix Paeoniae Rubra, Peach Kernel, Safflower, and Ligusticum chuanxiong were 119, 66, 189, 87, 189, and 125, respectively. Then, based on oral bioavailability ≥30% and drug-like ≥0.18, 103 compounds were screened out, including 20 of Astragalus Mongholicus, 2 of Angelica Sinensis, 29 of Radix Paeoniae Rubra, 23 of Peach Kernel, 22 of Safflower, and 7 of Ligusticum chuanxiong. Eighty-six compounds of “Earthworm” were obtained through literature reports,^[12]^ and 20 effective compounds were screened on the SwissADME (Swiss Institute of Bioinformatics) platform. Finally, we obtained a total of 82 effective ingredients by combining all the active ingredients and removing repetitive items. A total of 3107 targets were corresponding to the effective ingredients of these 7 drugs. The protein targets were standardized, and 666 unique protein targets were obtained after removing unrecognized and duplicate items.

### 3.2. Disease targets of LDH

**Figure 2.**
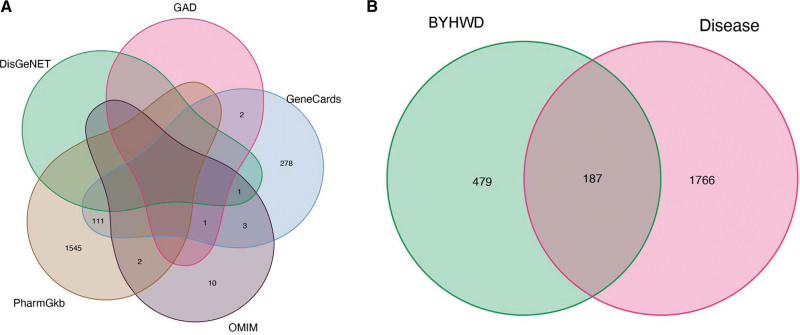
The Venn diagrams of targets. (A) The Venn diagram of LDH-related targets obtained by multi-database retrieval. (B) The Venn diagram of common targets of BYHWD and LDH. BYHWD = Buyang Huanwu decoction, LDH = lumbar disc herniation.

A number of 396, 17, 1658, 1, and 3 disease targets of LDH were obtained in the databases of Gene Cards, OMIM, Pharm GKB, DisGeNET, and GAD, respectively, and 1953 LDH-related targets were obtained after merging and de-duplication (Fig. 2A). After mapping the drug targets to the LDH targets, 187 common targets were obtained, such as PTGS2, NOS2, IL6, and MAPK1 (Fig. 2B).

### 3.3. Visualization network

**Figure 3.**
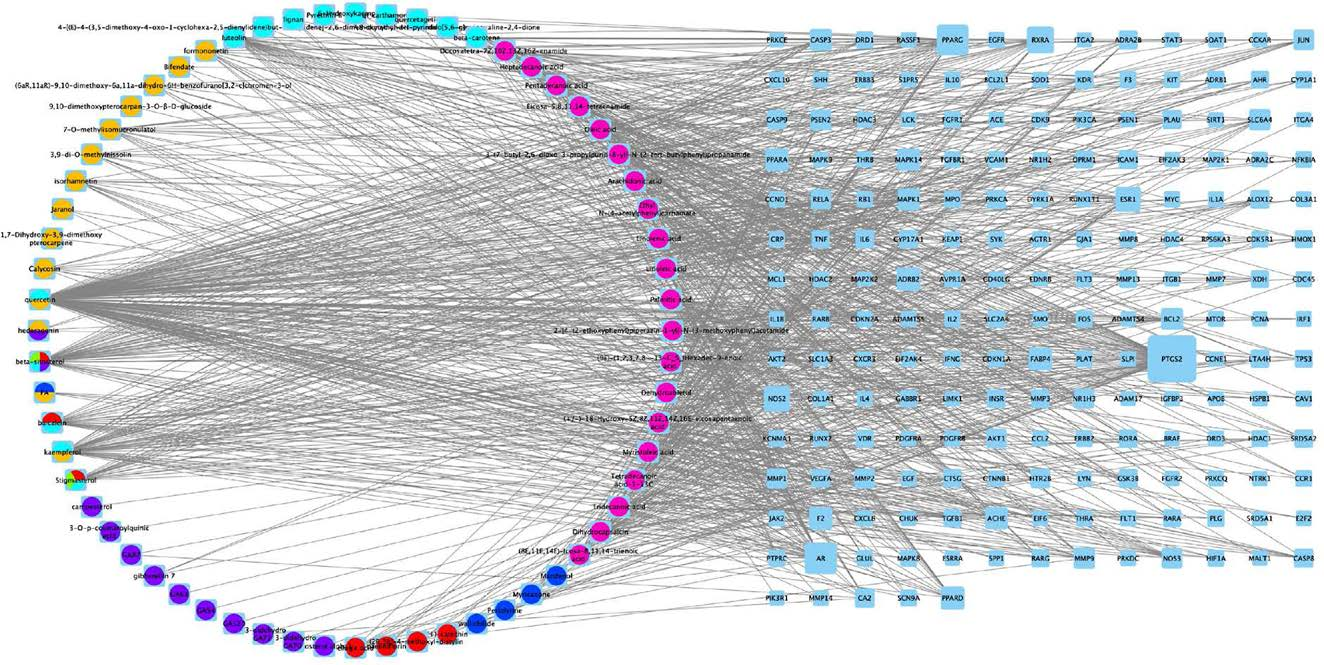
Visualization Network of “Drugs-Compounds-Targets”. Yellow represents Astragalus Mongholicus, green represents Angelica Sinensis, red represents Radix Paeoniae Rubra, dark blue represents Ligusticum chuanxiong, purple represents Peach Kernel, light blue represents Safflower, and purple represents Earthworm. The nodes of targets were presented according to the size of the degree.

We used Cytoscape to draw the relationship between the above active ingredients and targets. Two hundred fifty-one nodes and 959 relationship edges were obtained, and a visual network diagram of “Drugs-Compounds-Targets” was constructed. Compound nodes were presented as pie charts and different colors, and different colors represent that the compounds come from multiple Chinese herbs and drugs (Fig. 3).

### 3.4. PPI network

**Figure 4.**
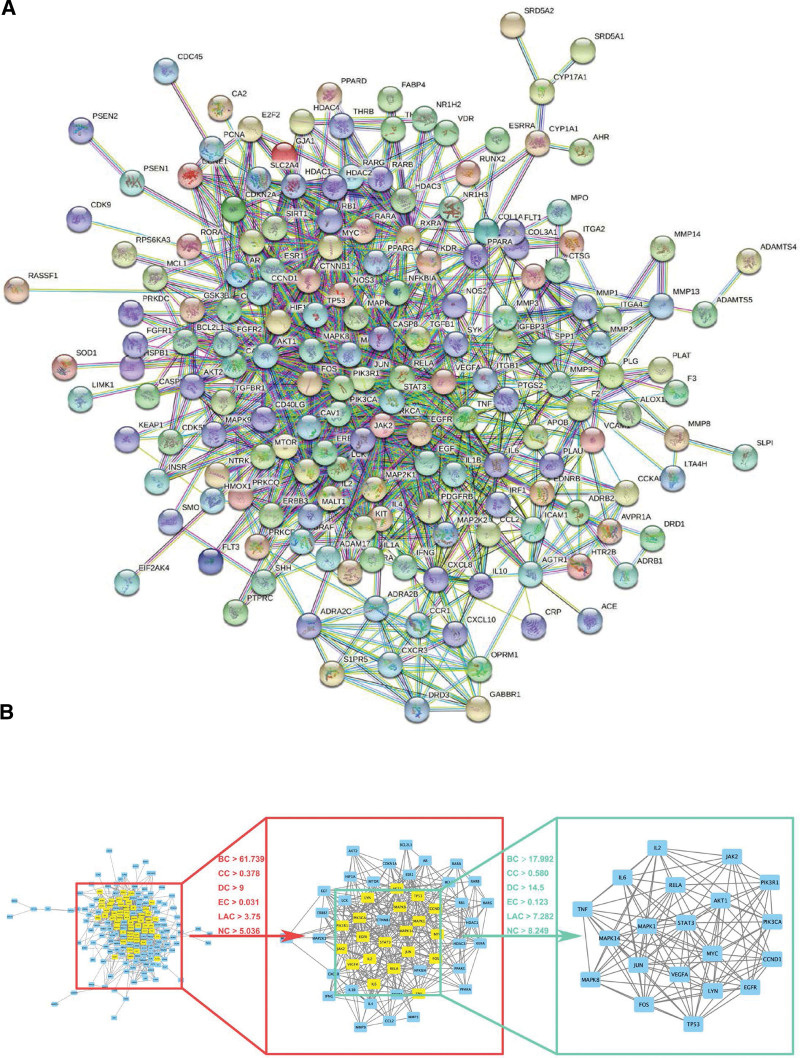
The PPI network and the core genes of it. (A) The PPI network. Nodes in the network represented different proteins. (B) The screening strategy for searching and obtaining core genes of PPI network. The initial screening criteria were BC > 61.739, CC > 0.378, DC > 9, EC > 0.031, LAC > 3.75, and NC > 5.036, as in the red box. The further filtering criteria were BC > 17.992, CC > 0.580, DC > 14.5, EC > 0.123, LAC > 7.282, and NC > 8.249, as in the blue box. BC = betweenness centrality, CC = closeness centrality, DC = degree centrality, EC = eigenvector centrality, LAC = local average connectivity, NC = network centrality, PPI = protein-protein interaction.

We uploaded the screened common protein targets to the STRING 11.0 platform and set the type as “homo sapiens”, then the confidence threshold was set as “the highest confidence (0.900)” according to the complexity of the network. We hid the disconnected nodes and kept other setting parameters unchanged, and ultimately constructed the PPI network diagram. The PPI network contains 187 proteins, 995 action edges, and 324 expected edges with enrichment *P* value <1.0e^−16^ (Fig. 4A).

### 3.5. Find core genes of PPI network

The above data files were imported into Cytoscape to generate a protein interaction network diagram, and the betweenness centrality, closeness centrality, degree centrality, eigenvector centrality, local average connectivity, and network centrality were calculated by CytoNCA network topology analysis plug-in, and finally, 175 nodes and 995 interaction edges were obtained. The nodes with the above indexes greater than the median of all gene nodes of each index were screened out, and a total of 52 nodes and 402 edges were obtained. Then the matrix was imported into Cytoscape to extract sub-networks, and 20 key nodes and 224 edges were calculated and screened again. Finally, the core network was extracted, and the nodes were key genes. The corresponding indexes were calculated in descending order of degree (Table 1). Therefore, it was predicted that STAT3, JUN, AKT1, MAPK1, and RELA in BYHWD were the key genes for treating LDH (Fig. 4B).

**Table 1:** Key genes of BYHWD in treatment of LDH.

Symbol	BC	CC	DC	EC	LAC	NC
STAT3	247.7163	0.761194	35	0.267751	11.6	27.05932
JUN	150.1542	0.71831	31	0.244457	11.48387	22.75809
AKT1	164.7936	0.689189	28	0.20868	9.5	18.63977
MAPK1	151.4275	0.68	27	0.204424	8.740741	16.32847
RELA	81.43702	0.653846	25	0.200093	9.6	15.53033
PIK3CA	86.99278	0.662338	25	0.19654	9.44	15.78613
TP53	83.96453	0.653846	24	0.189357	9.416667	15.56074
MAPK14	100.5109	0.653846	24	0.194405	8.166667	12.15381
PIK3R1	79.91766	0.653846	24	0.186653	9.083333	14.82169
TNF	68.7079	0.621951	21	0.165774	9.238095	13.39808
MAPK8	60.98472	0.621951	21	0.171864	7.52381	10.62076
EGFR	64.78361	0.621951	20	0.15813	8.1	11.51574
MYC	37.13057	0.621951	20	0.179781	9.4	11.88407
FOS	33.09109	0.614458	19	0.16928	9.157895	11.02425
JAK2	28.70425	0.607143	18	0.154071	8.777778	10.68858
IL2	20.35071	0.607143	18	0.168188	10.11111	12.16912
IL6	30.96351	0.593023	18	0.149575	8.888889	11.56662
VEGFA	36.45751	0.6	17	0.141457	7.529412	9.748016
LYN	23.8732	0.6	17	0.156981	8	9.273864
CCND1	19.72068	0.593023	16	0.143392	7.625	8.745377

### 3.6. GO function enrichment analysis

**Figure 5.**
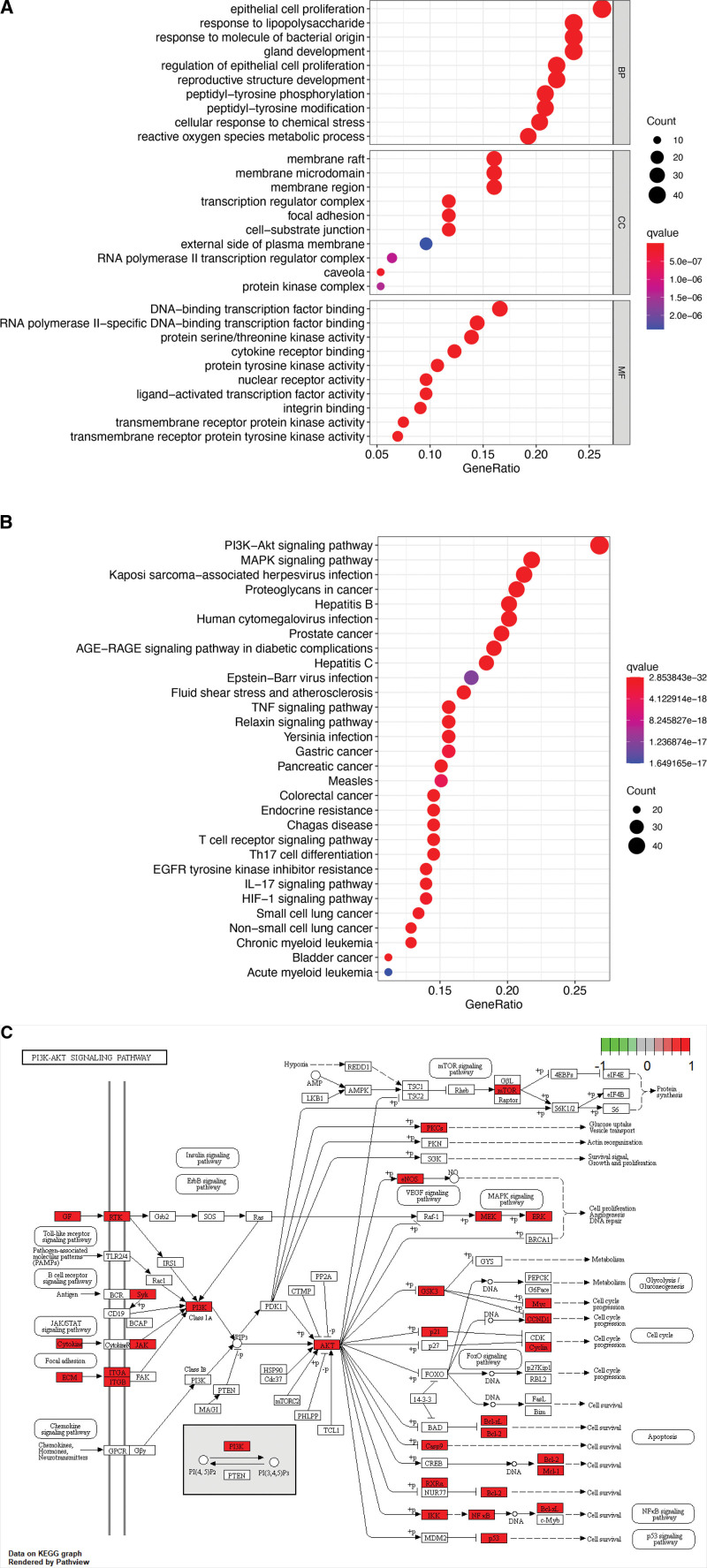
The results of the GO enrichment analysis and KEGG signaling pathway enrichment analysis. (A) The GO enrichment analysis of BYHWD candidate targets for LDH treatment. The horizontal axis of the bubble chart represents the gene ratio, the vertical axis represents the name of GO, and the corresponding bubble represents enriched gene counts. The smaller the *q* value, the redder the color, and the higher the significance of enrichment. (B) The KEGG analysis of candidate targets of BYHWD for LDH treatment. The horizontal axis of the bubble chart represents the gene ratio, the vertical axis represents the name of the signaling pathway, and the corresponding bubble represents enriched gene counts. The smaller the q value, the redder the color, and the higher the significance of enrichment. (C) The PI3K-AKT signaling pathway. BYHWD = Buyang Huanwu decoction, GO = Gene Ontology, KEGG = Kyoto Encyclopedia of Genes and Genomes, LDH = lumbar disc herniation.

GO enrichment analysis identified 3414 items of BYHWD in treating LDH and listed the top enrichment analysis results respectively (*q* value < 0.05). Its biological processes mainly include epithelial cell proliferation, response to lipopolysaccharide, response to bacterial molecules, gland development, and so on. Cell compounds mainly include membrane raft, membrane microdomain, membrane region, transcription regulatory complex, focal adhesion, and so on. Molecular functions mainly include DNA and RNA transcription factor binding, protease activity, cytokine receptor binding, nuclear receptor activity, transcription factor activity, etc (Fig. 5A).

### 3.7. KEGG signaling pathway enrichment analysis

The results of KEGG enrichment analysis showed that the key targets of BYHWD acting on LDH were mainly related to 173 signaling pathways such as PI3K-Akt signaling pathway, mitogen-activated protein kinase (MAPK) signaling pathway, hepatitis B, tumor necrosis factor (TNF) signaling pathway, interleukin (IL)-17 signaling pathway, etc. Thirty pathways with a large number of enriched genes were screened out, of which the PI3K-Akt signaling pathway was the most significantly enriched (Fig. 5B and C).

### 3.8. Molecular docking results

**Figure 6.**
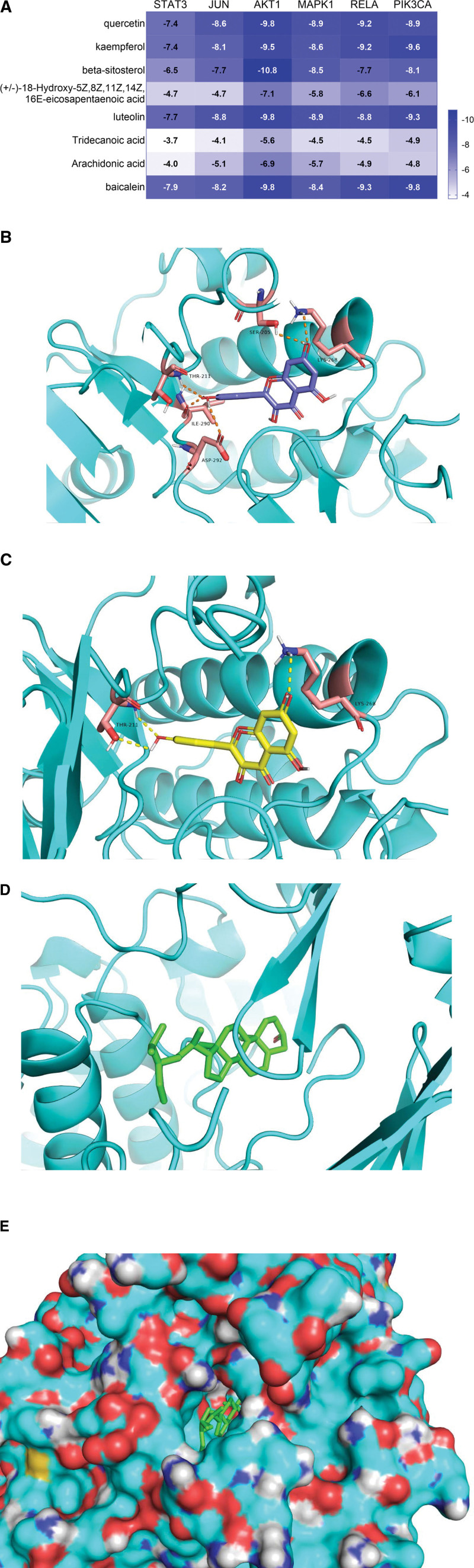
The heatmap of molecular docking results and molecular docking diagrams of core compounds and targets. (A) The molecular docking heatmap of core compounds and core targets of BYHWD. The dark and light colors represent the magnitude of the binding energy. The darker the blue color, the smaller the binding energy and the stronger the binding activity. (B) Quercetin and AKT1. (C) Kaempferol and AKT1. (D, E) Beta-sitosterol and AKT1. BYHWD = Buyang Huanwu decoction.

It is generally believed that the lower the energy is spent, the more likely the interaction will occur when the conformation of the ligand bound to the receptor is stabilized. The binding energy is less than –4.25 kcal mol^−1^, indicating that the ligand has a certain binding activity with the receptor. The binding activity is better when it is less than –5.0 kcal mol^−1^, and strong when it is less than –7.0 kcal mol^−1^.^[13]^ The top 8 active ingredients of the “Drugs-Compounds-Targets” network, including quercetin, kaempferol, beta-sitosterol, (+/–)-18-hydroxy-5Z, 8Z, 11Z, 14Z, 16E-eicosapentaenoic acid, luteolin, tridecanoic acid, arachidonic acid, and baicalein, were individually docked with the top 6 core protein targets, including STAT3, JUN, AKT1, MAPK1, RELA, and PIK3CA, and the binding energy threshold was set as less than –5.0 kcal mol^−1^. The results showed that quercetin, kaempferol, beta-sitosterol, luteolin, and baicalein were successfully docked to the corresponding proteins with good stability. Among them, the strongest binding was between beta-sitosterol and AKT1 (–10.8 kcal mol^−1^), and except the binding energy between beta-sitosterol and STAT3 was –6.5 kcal mol^−1^, all others had strong binding activity. This indicated that these compounds played a role through multiple targets. The binding activity of tridecanoic acid was the weakest in the overall level. Its binding activity with AKT1 was –5.6 kcal mol^−1^, which was stable, and the binding activities with other genes were greater than –5.0 kcal mol^−1^, which were relatively poor. All the compounds were successfully docked with AKT1 and had good binding activity (Fig. 6A). We selected the top 3 compounds including quercetin, kaempferol, and beta-sitosterol to visualize the docking results with AKT1 and output a 3-dimensional diagram of molecular docking (Fig. 6B–E).

## 4. Discussion

At present, the pathogenesis of LDH is gradually being recognized which includes mechanical compression of the nerve root, inflammatory stimulation of nerve root, apoptosis, and autoimmune mechanism of intervertebral disc.^[14–18]^ However, with the aggravation of the aging of the social population, the occurrence of LDH has a trend of younger age. Therefore, early detection and intervention treatment should be actively carried out. At present, conservative treatment mainly includes oral drug therapy, percutaneous block therapy, physical therapy, and exercise therapy.^[19,20]^ Among them, oral drugs such as non-steroidal anti-inflammatory drugs have better curative effects, but also have significant toxic and side effects such as gastrointestinal reactions and nephrotoxicity. The long-term application can lead to gastric mucosal erosion, gastric hemorrhage, peptic ulcer formation, and even perforation death. Recently, the advantages of Chinese herb are constantly being reflected, but it has the characteristics of multi-compounds, multi-targets, and multi-pathways, and all compounds work synergistically, so it is difficult to clarify the material basis and specific mechanism of action.

We constructed a “Drugs-Compounds-Targets” network and the above results showed that the effective components can improve LDH symptoms and the progress of intervention, including quercetin, kaempferol, beta-sitosterol, luteolin, and baicalein. Many recent studies have found that immune cells and immune factors produced by them were an important immunological mechanism of nerve injury in LDH.^[21–23]^ The expressions of inflammatory cytokine IL-1β, IL-6, and TNF-α were markedly increased in animal models of LDH, whereas these inflammatory cytokines have significant hyperalgesic effects. Quercetin reduces the production of inflammatory cytokines by inhibiting the MAPK/toll like receptor 4 signaling pathway and reduces the polarization of helper T cells 17 to exert anti-inflammatory effects.^[24]^ Nowadays, studies have found that kaempferol inhibits oxidative stress and neuroinflammation.^[25]^ The anti-inflammatory mechanisms of beta-sitosterol may differ from those of NSAIDs. It promotes cellular calcium uptake, inhibits the activity of myeloperoxidase and adenosine deaminase, and inhibits IL-1β, TNF-α levels.^[26,27]^ Luteolin is widely distributed in nature and numerous Chinese medicinal herbs contain this component, such as Lonicera japonica, Prunella vulgaris, and guar vegetables, which have anti-inflammatory, expectorant antitussive, antitumor, and antiviral pharmacological activities.^[28]^ Baicalein significantly suppressed the release of NO, IL-6, IL-1β, and TNF-α, thereby inhibiting inflammation occurrence.^[29]^ It has been demonstrated that baicalein can be converted to baicalin in vivo, which inhibits the production of inflammatory factor TNF-α, IL-1β, IL-6, IL-17, and matrix metalloproteinase-9, and regulates NF-κB signaling pathway to exert anti-inflammatory effects.^[30]^

In this study, 82 active compounds of BYHWD were excavated. A total of 3107 targets were corresponding to the effective components of the 7 drugs, and 666 unique protein targets were obtained after standardization by the Uniprot database. There were 187 possible targets for the treatment of LDH, of which 20 were core targets such as STAT3, JUN, AKT1, MAPK1, RELA, etc. The results of GO enrichment analysis showed that BYHWD participated in signal transduction, cell metabolism, DNA and RNA transcription, and other processes. As a signal transducer and activator of transcription, the activation of STAT3 is considered to be closely related to the development of chronic pain and participates in the pathway regulating the occurrence of intervertebral disc degeneration.^[31,32]^ The JUN gene is mainly involved in signal pathways such as apoptosis inhibition and neuronal degeneration.^[33,34]^ AKT1 is a core factor in the PI3K-Akt signaling pathway and plays an important role in cell growth, metabolic regulation, and cancer.^[35]^ MAPK1 is mainly involved in a variety of cellular processes, such as proliferation, differentiation, transcriptional regulation, and development.^[36]^ RELA is an important member of the NF-κB family. Its post-translational modification can accurately regulate the transcription activity of NF-κB and plays an important role in regulating important life activities such as inflammation, tumor, metabolism, and immune response.^[37]^ Therefore, it was speculated that BYHWD mainly alleviates the symptoms of patients by participating in cell metabolism, inflammatory reaction, transcriptional regulation, and immune response.

One hundred seventy-three signaling pathways were found by KEGG enrichment analysis. The pathways with higher gene ratio include PI3K-Akt signaling pathway, MAPK signaling pathway, virus infection-related pathway, tumor-related pathway, diabetic complication-related pathway, TNF signaling pathway, IL-17 signaling pathway, etc. Among them, the PI3K-Akt signaling pathway was the most significant enrichment. LDH has no significant correlation with tumor-related pathways and virus infection-related pathways, so we would not pay attention to them for the time being. IL-17A promotes intervertebral disc degeneration by inhibiting autophagy through activation of the PI3K/Akt/Bcl-2 signaling pathway.^[38]^ The MAPK pathway participates in central sensitization and nociceptive specific signaling in dorsal horn neurons.^[39]^ This study focuses on the PI3K-Akt signaling pathway. The PI3K-Akt signaling pathway is an important node in mammalian cells that controls cell growth, migration, proliferation, and metabolism, and plays an important role in regulating T cell development, function, and stability.^[40]^ The gene expression results studied by Tian et al show that hypoxia and nutritional deficiency can increase the relative expression of PI3K and Akt genes and inhibit the expression of functional genes.^[41]^ PI3K-Akt signaling pathway has a protective effect on mesenchymal stem cells derived from human nucleus pulposus against hypoxia and nutritional deficiency. When the PI3K-Akt pathway is blocked, it will aggravate intervertebral disc degeneration caused by inflammation, and inhibit the PI3K-Akt signal to promote osteogenic differentiation of mesenchymal stem cells in osteoporosis rat model.^[42,43]^ Moreover, the PI3K-Akt-mTOR signaling pathway is important for the normal metabolism of joint tissue.^[44]^ Inhibition of PI3K-Akt-mTOR signaling pathway leads to inhibition of fibroblast proliferation reduction, stimulation of apoptosis and autophagy, and provides new ideas for reducing epidural fibrosis caused by surgery.^[45]^ Xu et al^[46]^ found that LDH significantly increases thermo-mechanical abnormal pain and leads to degeneration of dorsal root ganglion (DRG) by inactivating the L-PGS/PI3K/Akt pathway. Upregulation of Nav1.6 can increase neuronal excitability and participate in neuropathic pain of DRG. By activating the TNF-/STAT3 pathway, histone H4 is superacetylated in the STAT3-mediated DRG Scn8a promoter region, resulting in L5-VRT-induced neuropathic pain.^[47]^

However, there are also deficiencies. First, the information of drug ingredients in databases and literature may still not be exhaustive, and insect drugs have a different research system than herbs. Then again, this study was only corroborated by relevant literature. Therefore, in a follow-up study, our group intends to intervene biological samples with full prescription drug delivery and decoupled drug delivery, and carry out high-throughput assays such as transcriptomics and proteomics to obtain the effect target profiles of BYHWD, to provide a high-quality data resource for more comprehensive annotation of the action mechanism of BYHWD.

## 5. Conclusions

In this study, we explored the mechanism, core targets, and signaling pathways of BYHWD in the treatment of LDH through network pharmacology and bioinformatics analysis. It was speculated that BYHWD plays a role in the treatment of LDH through multiple signaling pathways such as PI3K-Akt, MAPK, IL-17, and TNF, and PI3K-Akt signaling pathway may be one of the key mechanisms. These were conducive to optimize the experimental design, provide a reference for new drug development, and save costs to a certain extent. We also hope to promote BYHWD as a supplement and alternative treatment for LDH.

## Author contributions

GY wrote the paper. ZHJ, WXJ, ZSX, and LSJ performed the literature search and revised the paper. TPJ and LSJ guided the writing of the paper and reviewed the manuscript. All authors critically reviewed progressive drafts of the manuscript and approved the final version.

Methodology: Shanxing Zhang

Project administration: Shuaijie Lv

Resources: Shuaijie Lv

Supervision: Peijian Tong, Shuaijie Lv

Visualization: Xiaojian Wang, Xiaojian Wang,

Writing – original draft: Xiaojian Wang, Yong Gu

Writing – review & editing: Haijia Zhu

## References

[1] Wan ZY Zhang J Shan H. Epidemiology of lumbar degenerative phenotypes of children and adolescents: a large-scale imaging study. Global Spine J 2021;21925682211000707. 33843321 10.1177/21925682211000707 PMC10240608

[2] Arts MP Kuršumović A Miller LE. Comparison of treatments for lumbar disc herniation: systematic review with network meta-analysis. Medicine (Baltimore) 2019;98:e14410. 30762743 10.1097/MD.0000000000014410 PMC6408089

[3] Benzakour T Igoumenou V Mavrogenis AF Benzakour A. Current concepts for lumbar disc herniation. Int Orthop 2019;43:841–51. 30506088 10.1007/s00264-018-4247-6

[4] Du D. Treatment of prolapse of lumbar intervertebral disc by tuina massotherapy combined with oral administration of buyang huanwu tang—a report of 75 cases. J Tradit Chin Med 2007;27:43–5. 17393626

[5] Lin XJ Chen CY. Advances on study of treatment of lumbar disk herniation by Chinese medicinal herbs [in Chinese]. Zhongguo Zhong Yao Za Zhi 2007;32:186–91. 17432134

[6] Li S Zhang B. Traditional Chinese medicine network pharmacology: theory, methodology and application. Chin J Nat Med 2013;11:110–20. 23787177 10.1016/S1875-5364(13)60037-0

[7] Hopkins AL. Network pharmacology: the next paradigm in drug discovery. Nat Chem Biol 2008;4:682–90. 18936753 10.1038/nchembio.118

[8] Xu X Zhang W Huang C. A novel chemometric method for the prediction of human oral bioavailability. Int J Mol Sci 2012;13:6964–82. 22837674 10.3390/ijms13066964 PMC3397506

[9] Liu H Wang J Zhou W Wang Y Yang L. Systems approaches and polypharmacology for drug discovery from herbal medicines: an example using licorice. J Ethnopharmacol 2013;146:773–93. 23415946 10.1016/j.jep.2013.02.004

[10] Daina A Michielin O Zoete V. SwissTargetPrediction: updated data and new features for efficient prediction of protein targets of small molecules. Nucleic Acids Res 2019;47:W357–64. 31106366 10.1093/nar/gkz382 PMC6602486

[11] Daina A Michielin O Zoete V. SwissADME: a free web tool to evaluate pharmacokinetics, drug-likeness and medicinal chemistry friendliness of small molecules. Sci Rep 2017;7:42717. 28256516 10.1038/srep42717 PMC5335600

[12] Huang JW Gao HW Duan JF. Research on chemical composition and pharmacological effects of Geosaurus [in Chinese]. Guiding J Tradit Chin Med Pharm 2018;24:104–7.

[13] Hsin KY Ghosh S Kitano H. Combining machine learning systems and multiple docking simulation packages to improve docking prediction reliability for network pharmacology. PLoS One 2013;8:e83922. 24391846 10.1371/journal.pone.0083922 PMC3877102

[14] Shamji MF Jing L Chen J. Treatment of neuroinflammation by soluble tumor necrosis factor receptor type II fused to a thermally responsive carrier. J Neurosurg Spine 2008;9:221–8. 18764758 10.3171/SPI/2008/9/8/221 PMC2746856

[15] Shamji MF Allen KD So S. Gait abnormalities and inflammatory cytokines in an autologous nucleus pulposus model of radiculopathy. Spine (Phila Pa 1976) 2009;34:648–54. 19333095 10.1097/BRS.0b013e318197f013 PMC2712587

[16] Otoshi K Kikuchi S Konno S Sekiguchi M. The reactions of glial cells and endoneurial macrophages in the dorsal root ganglion and their contribution to pain-related behavior after application of nucleus pulposus onto the nerve root in rats. Spine (Phila Pa 1976) 2010;35:264–71. 20075775 10.1097/BRS.0b013e3181b8b04f

[17] Kim KW Kim YS Ha KY. An autocrine or paracrine Fas-mediated counterattack: a potential mechanism for apoptosis of notochordal cells in intact rat nucleus pulposus. Spine (Phila Pa 1976) 2005;30:1247–51. 15928547 10.1097/01.brs.0000164256.72241.75

[18] Fardon DF Milette PC. Nomenclature and classification of lumbar disc pathology. Spine (Phila Pa 1976) 2001;26:E93–113. 11242399 10.1097/00007632-200103010-00006

[19] Qaseem A Wilt TJ McLean RM Forciea MA; Clinical Guidelines Committee of the American College of P. Noninvasive treatments for acute, subacute, and chronic low back pain: a clinical practice guideline from the American College of Physicians. Ann Intern Med 2017;166:514–30. 28192789 10.7326/M16-2367

[20] Kankaanpaa M Taimela S Airaksinen O Hanninen O. The efficacy of active rehabilitation in chronic low back pain. Effect on pain intensity, self-experienced disability, and lumbar fatigability. Spine (Phila Pa 1976) 1999;24:1034–42. 10332798 10.1097/00007632-199905150-00019

[21] Zu B Pan H Zhang XJ Yin ZS. Serum levels of the inflammatory cytokines in patients with lumbar radicular pain due to disc herniation. Asian Spine J 2016;10:843–9. 27790311 10.4184/asj.2016.10.5.843 PMC5081318

[22] Pedersen LM Schistad E Jacobsen LM Roe C Gjerstad J. Serum levels of the pro-inflammatory interleukins 6 (IL-6) and -8 (IL-8) in patients with lumbar radicular pain due to disc herniation: a 12-month prospective study. Brain Behav Immun 2015;46:132–6. 25653193 10.1016/j.bbi.2015.01.008

[23] Elkan P Sten-Linder M Hedlund R Willers U Ponzer S Gerdhem P. Markers of inflammation and fibrinolysis in relation to outcome after surgery for lumbar disc herniation. A prospective study on 177 patients. Eur Spine J 2016;25:186–91. 25962814 10.1007/s00586-015-3998-7

[24] Mahmoud Hashemi A Solahaye Kahnamouii S Aghajani H. Quercetin decreases Th17 production by down-regulation of MAPK-TLR4 signaling pathway on T cells in dental pulpitis. J Dent (Shiraz) 2018;19:259–64. 30680297 PMC6338691

[25] Kouhestani S Jafari A Babaei P. Kaempferol attenuates cognitive deficit via regulating oxidative stress and neuroinflammation in an ovariectomized rat model of sporadic dementia. Neural Regen Res 2018;13:1827–32. 30136699 10.4103/1673-5374.238714 PMC6128063

[26] Liz R Zanatta L dos Reis GO. Acute effect of β-sitosterol on calcium uptake mediates anti-inflammatory effect in murine activated neutrophils. J Pharm Pharmacol 2013;65:115–22. 23215694 10.1111/j.2042-7158.2012.01568.x

[27] Liao PC Lai MH Hsu KP. Identification of β-sitosterol as in vitro anti-inflammatory constituent in Moringa oleifera. J Agric Food Chem 2018;66:10748–59. 30280897 10.1021/acs.jafc.8b04555

[28] Baek Y Lee MN Wu D Pae M. Luteolin reduces adipose tissue macrophage inflammation and insulin resistance in postmenopausal obese mice. J Nutr Biochem 2019;71:72–81. 31302373 10.1016/j.jnutbio.2019.06.002

[29] Duan DD Wang KX Zhou YZ Qin XM Gao L Du GH. Baicalein exerts beneficial effects in d-galactose-induced aging rats through attenuation of inflammation and metabolic dysfunction. Rejuvenation Res 2017;20:506–16. 28548620 10.1089/rej.2017.1919

[30] Dinda B Dinda S DasSharma S Banik R Chakraborty A Dinda M. Therapeutic potentials of baicalin and its aglycone, baicalein against inflammatory disorders. Eur J Med Chem 2017;131:68–80. 28288320 10.1016/j.ejmech.2017.03.004

[31] Zhou T Lin H Cheng Z Ji C Zhang C Tian J. Mechanism of microRNA-146a-mediated IL-6/STAT3 signaling in lumbar intervertebral disc degeneration. Exp Ther Med 2017;14:1131–5. 28810568 10.3892/etm.2017.4611 PMC5525655

[32] Xue ZJ Shen L Wang ZY Hui SY Huang YG Ma C. STAT3 inhibitor WP1066 as a novel therapeutic agent for bCCI neuropathic pain rats. Brain Res 2014;1583:79–88. 25084036 10.1016/j.brainres.2014.07.015

[33] Wang A Jiang H Liu Y. Rhein induces liver cancer cells apoptosis via activating ROS-dependent JNK/Jun/caspase-3 signaling pathway. J Cancer 2020;11:500–7. 31897245 10.7150/jca.30381 PMC6930441

[34] Schenkel J. Activation of the c-Jun transcription factor following neurodegeneration in vivo. Neurosci Lett 2004;361:36–9. 15135887 10.1016/j.neulet.2003.12.011

[35] Cole PA Chu N Salguero AL Bae H. AKTivation mechanisms. Curr Opin Struct Biol 2019;59:47–53. 30901610 10.1016/j.sbi.2019.02.004 PMC6752985

[36] Javan GT Salhotra A Finley SJ Soni S. Erythroblast macrophage protein (Emp): past, present, and future. Eur J Haematol 2018;100:3–9. 10.1111/ejh.12983 29032607

[37] Lu X Yarbrough WG. Negative regulation of RelA phosphorylation: emerging players and their roles in cancer. Cytokine Growth Factor Rev 2015;26:7–13. 25438737 10.1016/j.cytogfr.2014.09.003

[38] He WS Zou MX Yan YG. Interleukin-17A promotes human disc degeneration by inhibiting autophagy through the activation of the phosphatidylinositol 3-kinase/Akt/Bcl2 signaling pathway. World Neurosurg 2020;143:e215–23. 32712400 10.1016/j.wneu.2020.07.117

[39] Ji RR Gereau RW Malcangio M Strichartz GR. MAP kinase and pain. Brain Res Rev 2009;60:135–48. 19150373 10.1016/j.brainresrev.2008.12.011 PMC2666786

[40] Pompura SL Dominguez-Villar M. The PI3K/AKT signaling pathway in regulatory T-cell development, stability, and function. J Leukoc Biol 2018; doi: 10.1002/JLB.2MIR0817-349R. 10.1002/JLB.2MIR0817-349R 29357116

[41] Tian D Liu J Chen L Zhu B Jing J. The protective effects of PI3K/Akt pathway on human nucleus pulposus mesenchymal stem cells against hypoxia and nutrition deficiency. J Orthop Surg Res 2020;15:29. 31992313 10.1186/s13018-020-1551-9 PMC6988348

[42] Tan FZ Dai HL. TAZ accelerates osteogenesis differentiation of mesenchymal stem cells via targeting PI3K/Akt. Eur Rev Med Pharmacol Sci 2019;23(Suppl 3):81–8. 31389578 10.26355/eurrev_201908_18633

[43] Deng X Wu W Liang H. Icariin prevents IL-1beta-induced apoptosis in human nucleus pulposus via the PI3K/AKT pathway. Evid Based Complement Alternat Med 2017;2017:2198323. 29259641 10.1155/2017/2198323 PMC5702406

[44] Sun K Luo J Guo J Yao X Jing X Guo F. The PI3K/AKT/mTOR signaling pathway in osteoarthritis: a narrative review. Osteoarthritis Cartilage 2020;28:400–9. 32081707 10.1016/j.joca.2020.02.027

[45] Dai J Sun Y Chen D. Negative regulation of PI3K/AKT/mTOR axis regulates fibroblast proliferation, apoptosis and autophagy play a vital role in triptolide-induced epidural fibrosis reduction. Eur J Pharmacol 2019;864:172724. 31600493 10.1016/j.ejphar.2019.172724

[46] Xu W Ding W Sheng H Lu D Xu X Xu B. Dexamethasone suppresses radicular pain through targeting the L-PGDS/PI3K/Akt pathway in rats with lumbar disc herniation. Pain Pract 2021;21:64–74. 32640501 10.1111/papr.12934

[47] Ding HH Zhang SB Lv YY. TNF-alpha/STAT3 pathway epigenetically upregulates Nav1.6 expression in DRG and contributes to neuropathic pain induced by L5-VRT. J Neuroinflammation 2019;16:29. 30736806 10.1186/s12974-019-1421-8 PMC6368780

